# Evaluation of novice reader diagnostic performance in coronary CT angiography using an advanced cardiac software package

**DOI:** 10.1007/s11548-013-0953-0

**Published:** 2013-11-08

**Authors:** Peter Dankerl, Matthias Hammon, Alexey Tsymbal, Alexander Cavallaro, Stephan Achenbach, Michael Uder, Rolf Janka

**Affiliations:** 1 Department of Radiology, University Hospital Erlangen, Maximiliansplatz 1, 91054 Erlangen, Germany; 2 Department of Cardiology, University Hospital Erlangen, Ulmenweg 18, 91054 Erlangen, Germany; 3 Corporate Technology, Imaging and Computer Vision Department, Siemens AG, San-Carlos Str. 7, 91054 Erlangen, Germany

**Keywords:** Coronary computed tomography angiography, Coronary artery stenosis, Advanced cardiac software package

## Abstract

**Purpose:**

The purpose of this research was to evaluate whether a commercially available advanced cardiac software package for coronary CT angiography (CTA) interpretation may reliably assist inexperienced readers to screen for significant coronary artery stenoses.

**Methods:**

Coronary CTA data sets of 61 consecutive patients with suspected coronary artery disease were evaluated by three novice readers with no experience in cardiac CT interpretation. In the first 15 patients, the novice readers were trained to use the advanced cardiac software package (includes automatic detection of coronary vessels, curved MPR and VRT reconstructions and a measurement too) knowing the results of an expert read. In the next 46 patients, the novice readers had to state whether there is a significant coronary artery stenosis ($>$50 %) and if they are confident with their diagnosis. The results of the novice readers were compared to the expert read.

**Results:**

The 46 coronary CTA data sets contained 184 vessels with 15 stenoses in 9 patients. On a per-vessel analysis, novice reader 1/2/3 demonstrated 60 %/100 %/ 93% sensitivity, and 98 %/90 %/86 % specificity. Per patient, the readers diagnosed 36/28/29 cases correctly as free of stenoses, 6/9/8 correctly as having at least one stenosis, missed 3/0/1 cases with a stenosis and overdiagnosed 1/9/8 patients. Cohen’s kappa values for the three readers versus the expert were 0.60, 0.61 and 0.54. The three novice readers felt confident in the diagnosis of 36/33/30 patients. In these patients, they missed one significant stenosis, showed a sensitivity of 100 %/100 %/75 % and a specificity of 100 %/92 %/88 %.

**Conclusions:**

The evaluated advanced cardiac software package successfully assists novice readers in interpreting coronary CTA data sets especially in ruling out significant coronary artery stenosis.

## Introduction

Coronary computed tomography angiography (coronary CTA) can be used to identify and rule out coronary artery stenoses in selected patients [1–3]. In recent years, the number of coronary CTA studies has steadily increased for various reasons. More and more hospitals and radiological practices have access to multi-detector computed tomography systems which permit coronary CT imaging [4]. Furthermore, radiation exposure for routine coronary CTA can be reduced to less than 1 mSv per examination [5, 6]. The majority of patients prefer CT to invasive coronary angiography [7], and an increasing amount of clinical data, for example regarding the use of coronary CTA in acute chest pain patients, demonstrates the method’s efficacy [8].

However, the accurate identification of significant coronary stenoses is a challenging task that requires extensive training and experience, and according to Saur [9] is often constrained by the evaluator’s knowledge and ability. Therefore, the American College of Cardiology and American Heart Association have developed clinical competence criteria to standardize training for interpretation of coronary CTA [10]. Expertise and experience are measured in competence levels (1–3), whereas level 1 can be achieved during a one month training and interpretation of 50 coronary CTA examinations—while level 3 requires 6 month of training, 300 interpreted examinations as well as ongoing teaching and research in the area of coronary CTA. However, some authors [11] claim that the learning curve is substantially longer than suggested by the competence criteria. As indicated by the 15-fold increase in research on cardiac CT between 1996 and 2006 [12], most likely the number of coronary CTA studies dramatically increased, outweighing the number of expert readers.

The purpose of this research was to evaluate whether commercially available advanced cardiac software package for coronary CTA interpretation may reliably assist inexperienced readers to screen for significant coronary artery stenoses.

## Materials and methods

The institutional review board approved this study and waived the need for informed consent.

### Patient acquisition and scan technique

Data sets from 61 consecutive patients undergoing clinically indicated routine coronary CTA (24 male, 37 female; mean age $54.3\pm 9.4$ years was retrospectively included for evaluation. Multidetector CT had been performed with a 128-section dual source CT system (Somatom Flash$^\circledR$, Siemens, Forchheim, Germany). Clinical indications for coronary CTA included 3 patients prior and 5 after aortic valve replacement, 3 before coronary bypass operation and 50 patients with chest pain and intermediate pretest likelihood of coronary artery disease. The patients’ mean body weight was $84.7 \pm 12.8$ kg. Patients with a heart rate greater than 60 beats/min before the examination received oral administration of a $\beta$-blocker (atenolol, 100 mg) 1 h prior to the scan. During coronary CTA, the patients’ mean heart rate was $65.9 \pm 13.6$. Adopted doses of 50–80 ml i.v. contrast agent (350 mg iodine/ml, Imeron$^\circledR$, Bracco, Friedrichshafen, Germany), followed by 50 ml of saline solution were administered to all patients at a flow rate of 4–7 ml/s (54 patients received 60 ml, 5 patients 50 ml and 2 patients 80 ml contrast media). Images were acquired in prospectively ECG-triggered high-pitch spiral mode, and acquisition was timed to start at 60 % of the patients’ R-peak to R-peak interval [13]. Tube voltage was 100 kV, tube current 320 mAs per rotation. Reconstructed slice thickness was 0.6 mm, slice increment 0.3 mm, and a 3D adaptive noise reduction algorithm termed B26 kernel by the manufacturer [14] was applied.

### Advanced cardiac software package

**Fig. 1:**
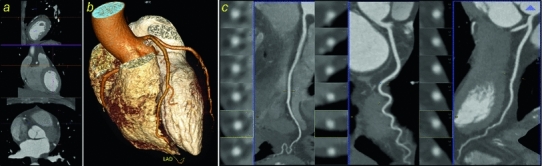
The advanced cardiac software package consists of: **a** An interactive 3D multiplanar viewer. **b** Automatically identified, segmented and named right coronary artery (RCA), circumflex coronary artery (CX), and left anterior descending coronary artery (LAD) displayed as 3D volume rendering technique (VRT) reconstruction. **c** Curved multiplanar reformations (MPR) of all three coronary arteries (RCA, CX and LAD) are automatically generated and true axial views are provided. The software enables real-time navigation between corresponding locations in the 3D interactive multiplanar views, the vessels’ curved MPRs and the true axial views

**Fig. 2:**
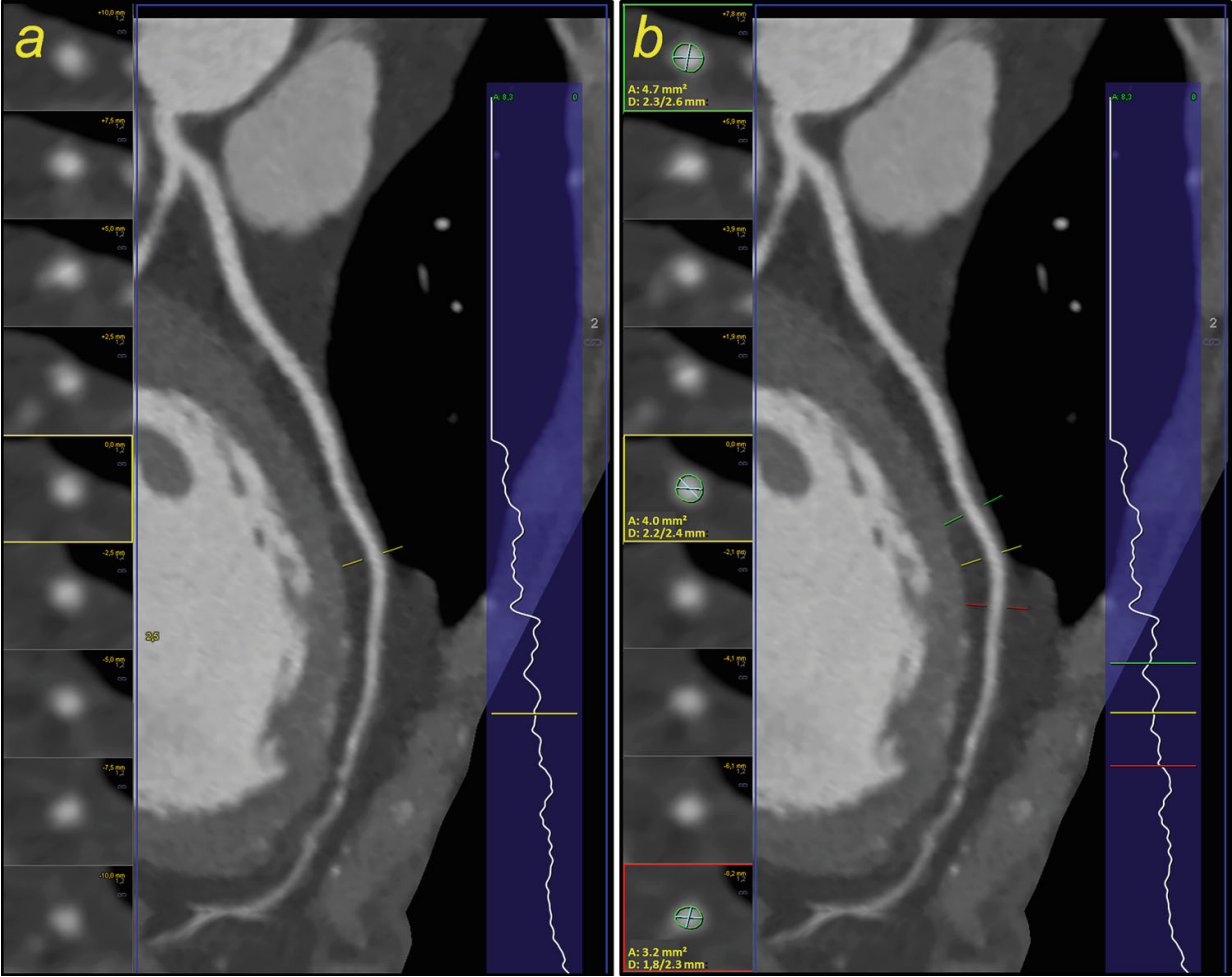
**a** The advanced cardiac software package indicates a stenosis by displaying a vertical-section profile from the curved multiplanar reformations for each coronary vessel, here the left anterior descending coronary artery (LAD). **b** One feature allows stenosis quantification by three adjustable bars, which are aligned orthogonally to the vessel direction (true axial) and are projected onto the curved MPR and the vertical-section profile. At the location of each bar, the diameter for the cross section of contrasted vessel is measured. By positioning the adjustable bars in accordance with the vertical-section profile of a vessel, for instance proximal, in and distal the narrowest part of a stenotic vessel segment, it calculates the degree of stenosis both with respect to the diameter and area of the remaining lumen. Furthermore, the axial view (in the orthogonal direction to the vessel) at the position of each bar (including the measured values) is displayed

We investigated a commercially available advanced cardiac software package (syngo.via$^\circledR$, Siemens, Forchheim, Germany). It semi-automatically reformats the original images, segments the coronary arteries and provides a measurement tool for stenosis quantification (Figs. 1, 2).

### Image evaluation

#### Standard of reference

A consensus read of two experienced readers (5 and 6 years experience) and one expert reader (15 years of experience in cardiac CT and Electron-Beam Tomography) supplied the reference standard. Therefore, they evaluated all 61 data sets for the presence of a significant coronary artery stenosis ($>$50 % reduction in diameter) in the left main (LM), the left anterior descending (LAD), the left circumflex (CX) and the right coronary artery (RCA), utilizing a standard 3D MPR visualization software (Leonardo, Siemens, Forchheim, Germany).

#### Readers

Three novice readers (medical students in their last semester of medical school), inexperienced in cardiac imaging and CT interpretation, were trained in the use of the advanced cardiac software package. Under the supervision of a radiologist experienced in using the advanced cardiac software package, the students interpreted the examinations of the first 15 patients applying the software and therefore were aware of the expert’s interpretation.

#### Workflow of image evaluation applying the advanced cardiac software package

The CT data sets were transferred to a 3D workstation and automatically post-processed by the advanced cardiac software package. For the experimental read, the three novice readers independently interpreted the same 46 examinations without further assistance and they were blinded to the patients’ clinical data. The workflow demanded the readers to apply the advanced cardiac software package detect stenoses and use the stenosis quantification tool. If a stenosis seemed to be overestimated by the advanced cardiac software package, for example due to artifacts, they were encouraged to use the assisted navigation to re-examine the stenosis in the corresponding location in multiplanar views. If in doubt, readers were instructed to base their final diagnosis on their perception of vessel stenosis rather than on the system’s calculated degree of severity, using the software as assistance, but leaving the final diagnosis up to the human readers. Furthermore, the readers were asked to document their confidence about their diagnosis for each vessel (confident/not confident).

### Statistical analysis

The statistical analysis on the 46 test patients was calculated on a per-vessel view and on a per-patient view for all three novice readers.

#### Per vessel

True positives (TP), false positives (FP), true negatives (TN), false negatives (FN), sensitivity, specificity, negative (NPV) and positive predictive values (PPV), as well as diagnostic odds ratio (DOR) and interreader agreement of the novice reader and the expert reader (Cohen’s kappa and McNemar test) were analyzed.

#### Per patient

TP, FP, TN, FN, sensitivity, specificity, NPV, PPV were calculated.

Additionally, the readers’ per-patient diagnostic performance (sensitivity, specificity, PPV, NPV) depending on their diagnostic confidence were evaluated.

## Results

### Standard of reference

The 15 training coronary CTA data sets consisted of 60 evaluable coronary arteries, of which 7 vessels in 6 patients revealed significant stenoses (0/LM, 4/LAD, 1/CX, 2/RCA).

The 46 experimental coronary CTA data sets contained 184 evaluable coronary arteries, 169 without, and 15 with significant stenoses (0/LM, 6/LAD, 4/CX, 5/RCA). On a per-patient basis, 37 patients had no stenoses, while 9 patients showed at least one significant coronary artery stenosis. No coronary CTA data set was excluded due to artifacts or insufficient image quality.

### Performance of the novice readers

The readers where able to process all 46 investigated coronary CTA data sets applying the advanced cardiac software package. No data set had to be excluded from the experiment due to artifacts or insufficient image quality.

On a per-vessel analysis, the three novice readers (reader 1/2/3) demonstrated 4/16/23 false positives, 6/0/1 false negatives and correctly identified 165/153/146 vessels as nonstenotic. This resulted in a sensitivity of 60 %/100 %/93 % and a specificity of 98 %/90 %/86 % and a diagnostic odds ratio (DOR) of 62, 145 and 89. Displaying a Cohen’s kappa of 0.61, reader 2 demonstrated substantial interreader agreement with the reference standard [15], while readers 1 and 3 demonstrated good agreement (0.60/0.54) [16]. McNemar’s p-value did not indicate a significant difference between the diagnostic performances of reader 1 and the reference standard ($p=0.75$) (Table 1).

**Table 1:** Diagnostic performance of all three novice readers in detecting significant coronary artery stenoses compared to the expert read (=reference standard)—per-vessel analysis

	Reader 1	Reader 2	Reader 3
True positives *reference standard n* $=$15	9	15	14
True negatives *reference standard n* $=$169	165	153	146
False negatives	6	0	1
False positives	4	16	23
Sensitivity (%)	60	100	93
Specificity (%)	98	90	86
Positive predictive value (%)	69	48	36
Negative predictive value (%)	97	100	99
Diagnostic odds ratio (DOR)	61.9	144.9	88.9
Cohen‘s kappa	0.60	0.61	0.54
McNemar $p$-value	0.75	$<\!0.001$	$<\!0.001$

Per patient, the readers diagnosed 36/28/29 cases correctly as free of stenoses, 6/9/8 correctly as having at least one significant stenosis, missed 3/0/1 cases with a significant stenosis and overdiagnosed 1/9/8 patients. On the per-patient basis, sensitivity was 67 %/100 %/89 % and specificity 97 %/76 %/78 % (Table 2).

**Table 2:** Diagnostic performance of all three novice readers in detecting significant coronary artery stenoses compared to the expert read (=reference standard)—per-patient analysis

	Reader 1	Reader 2	Reader 3
True positives *reference standard* $n=9$	6	9	8
True negatives *reference standard* $n=37$	36	28	29
False negatives (%)	3	0	1
False positives (%)	1	9	8
Sensitivity (%)	67	100	89
Specificity (%)	97	76	78
Positive predictive value (%)	86	50	50
Negative predictive value (%)	92	100	97
Mean false positives per patient	0.09	0.37	0.5

**Fig. 3:**
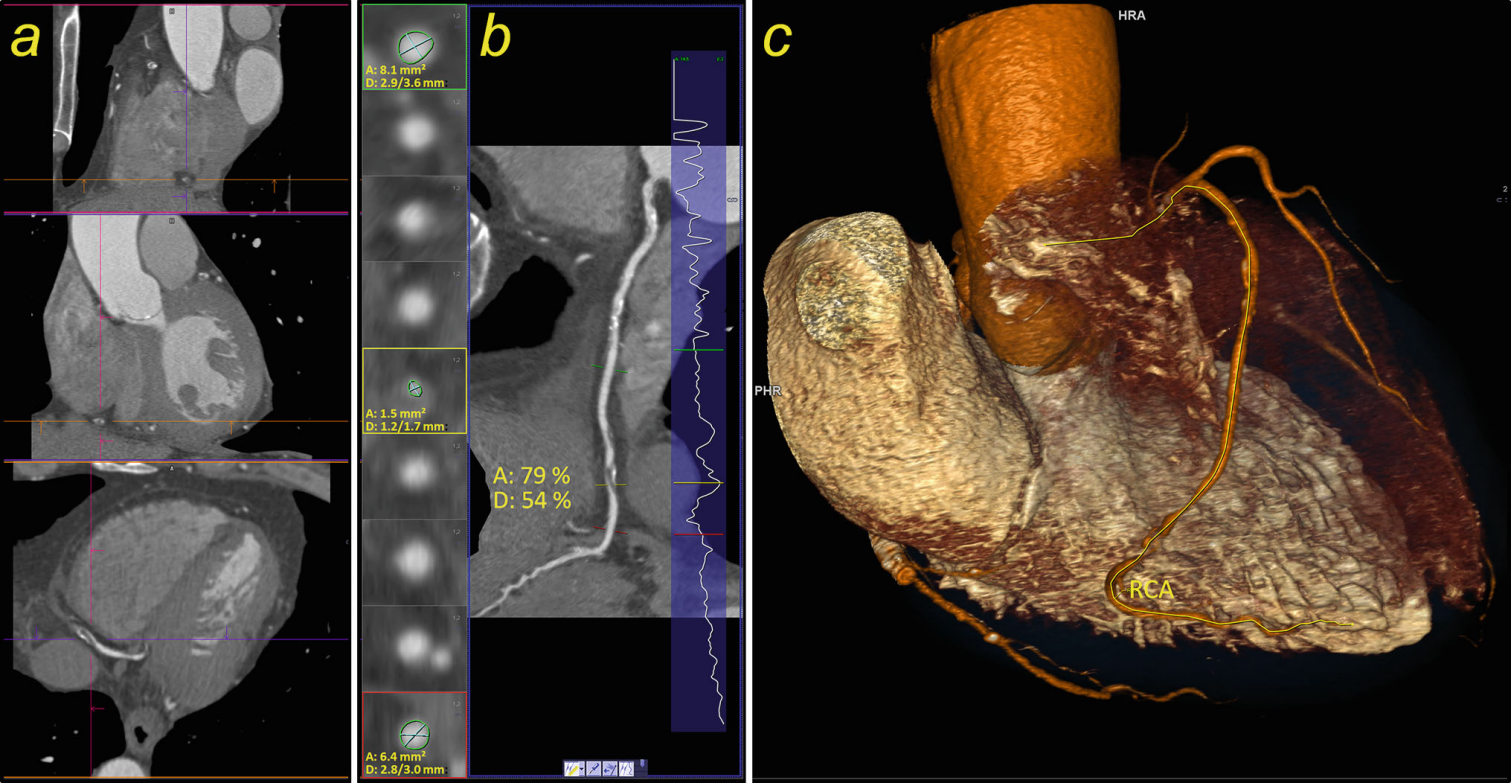
Example of a significant stenosis in the right coronary artery (RCA) as displayed by the advanced cardiac software package. **a** Interactive multiplanar reformations (sagittal, coronal and axial plane). **b** Curved multiplanar reformation and the vertical-section profile indicating lumen’s diameter (*right*). The true axial views are shown proximal, in and distal the narrowest part of a stenotic vessel segment and degree of stenosis with respect to the diameter (D) and area (A) of the remaining lumen is calculated (*left*) **c** 3D volume rendering technique (VRT) reconstruction. The significant stenosis was correctly diagnosed by two of the three novice readers

Overall readers felt uncertain about their diagnosis in 39/138 patients (28 %). In these cases, the sensitivity, specificity, PPV and NPV for detection of patients with significant coronary stenosis ranged between 50 and 63 %. If the readers felt certain about their diagnosis (99/138 patients), sensitivity/specificity/PPV/NPV increased to 93 %/95 %/76 %/99 %. They correctly interpreted 80/99 cases as free of stenosis and missed one significant stenosis in 99 patients (Table 3, Fig. 3).

**Table 3:** Novice readers’ diagnostic performance in detecting significant coronary artery stenoses depending on their diagnostic confidence—per-patient analysis

	Confident				Nonconfident			
Reader	1	2	3	$\sum$	1	2	3	$\sum$
True positives	2	8	3	13	4	1	5	10
True negatives	34	23	23	80	2	5	5	12
False positives	0	2	3	5	1	0	6	7
False negatives	0	0	1	1	3	7	0	10
$\sum$	36	33	30	99	10	13	16	39
Sensitivity (%)	100	100	75	93	57	13	100	50
Specificity (%)	100	92	88	95	67	100	45	63
Positive predictive value (%)	100	80	50	76	80	100	45	59
Negative predictive value (%)	100	100	96	99	40	42	100	55

## Discussion

As demonstrated by this work, novice readers revealed moderate sensitivity and specificity in detecting significant coronary artery stenosis when using a commercially available advanced cardiac software package. However, when taking readers’ confidence level into consideration, novices displayed excellent sensitivity and NPV for detection of significant coronary artery stenoses. The presented workflow considers the advanced cardiac software as a tool inexperienced readers (e.g., interns) can apply to screen for significant stenoses in coronary CTA. If the expert would have reevaluated only cases which were marked as diseased or uncertain by the novice readers which corresponded to 40 % of all cases, he would have missed only one significant stenosis. Our results stand in accordance with the papers of Meyer et al. and Arnoldi et al. [17, 18], who demonstrated computer-aided systems to be helpful for detection of coronary stenosis by less-experienced readers. In contrast to our work, the software was applied as second reader, whereas the setup in our experiment simulates utilization of the software in the initial read. Additionally, in our experiment, the software never suggested a diagnosis. Instead, in the presented study, inexperienced readers apply the advanced cardiac software package as semi-automatic coronary artery visualization and segmentation tool, with the final decision up to the human interpreter. Our work stands in consistency with Goldenberg et al. [19] who propose a CADx as an initial analyzer (rather than a second reader), but leaves the final decision to the physician. As displayed by the excellent sensitivity and NPV, as well as the acceptable specificity and PPV, the presented method fulfills demands of a screening test [20].

According to Anders et al. and Ferencik et al. [21, 22], the diagnostic accuracy of prerendered images, for example curved MPR and VRT, is inferior to conventional interactive reading of coronary CTA data. However, the investigated software offers interactive 3D multiplanar view and prerendered images, as well as real-time navigation between corresponding locations from the prerendered images and the interactive 3D multiplanar viewer. This provides the speed and convenience of post-processed images and the precision of interactive 3D multiplanar reading.

Pugliese et al. [11] studied the learning curve of members of a cCTA fellowship. They also investigated novices in reading coronary CTA. However, since all readers were radiology/cardiology fellows, in contrast to our study, they all had at least some level of experience in reading CT images or diagnosing coronary artery disease. After one year of fellowship, investigated readers reached competence level 3 for the number of interpreted examinations [10]. Although the results from [11] (naturally using different data sets), and our study (due to different experimental protocols, varying study hypotheses, distinct analyzing approaches, etc.) have to be compared very carefully and do not display a perfect concordance, we might compare the diagnostic odds ratio (DOR), a single and powerful indicator of test performance [23]. Even without considering level of confidence for our readers, they demonstrated a higher DOR (62, 145, 89) compared to [11] (between 15 and 27). Reported sensitivity (68–75 %) and specificity (88–92 %), both after one year of training were similar or worse to the overall performance of our readers.

Further, novices’ diagnostic performance is validated by Cohen’s kappa, attesting moderate to good interreader agreement to the reference standard. McNemar’s value suggests high correlation between reader 1 and the reference standard. However, the need for dedicated and structured training in interpreting coronary CTA cannot be overemphasized.

One limitation of our study is that the novice readers did not initially analyze the coronary CTA data sets without the assistance of the advanced cardiac software. So we do not know if and how much the advanced software package has increased the diagnostic performance of the novice readers. Another limitation is that the expert reading was not verified by invasive coronary angiography and significant CTA stenoses could not be verified to be hemodynamically relevant or insignificant. It is a well-known fact that coronary CTA has a limited positive predictive value, i.e., too many patients are incorrectly suspected of having a significant coronary artery stenosis [24]. However, the aim of our research was mainly set on comparing the interpretation results of the novices with an expert read. The clinical effect of coronary CTA stenosis possibly displaying hemodynamic significance could be the next step in future work. Moreover, our study focused on the evaluation of consecutive, unselected routine coronary CTA data sets. Most investigated patients presented with chest pain and intermediate pretest likelihood of coronary artery disease and after negative CTA did not receive further invasive coronary angiography. Finally, the patient number was relatively small and the number of diseased arteries relatively low. This low incidence, however, represents a clinical cohort of patients with an intermediate pretest likelihood of coronary artery disease better than a preselected group of diseased patients. Although coronary artery disease with 20 % accounts for the leading cause of death in the western world, our incidence indeed is way higher than the average 30 % lifetime prevalence for men and 15 % for women in Western countries [25].

## Conclusions

The applied advanced cardiac software successfully assists novice readers in interpreting coronary CTA data sets. Evaluated software and displayed manner of use demonstrates moderate sensitivity and specificity for novice readers. When further taking readers’ confidence level into account, sensitivity of detecting coronary stenosis was excellent. Therefore, the advanced cardiac software may be useful for novice readers as an initial analysis tool for screening and ruling out significant stenoses in coronary CT angiography.
